# The Endovascular Embolization of an Isolated Internal Iliac Artery Aneurysm: A Case Report

**DOI:** 10.7759/cureus.64330

**Published:** 2024-07-11

**Authors:** Mohamed M Abdelhadi, Martin Maresch, Ahmed I Elmasry

**Affiliations:** 1 Vascular Surgery, Royal Medical Services-Military Hospital, Riffa, BHR; 2 General Surgery, Royal Medical Services, Riffa, BHR

**Keywords:** aneurysm coiling, internal iliac artery aneurysm, isolated internal iliac aneurysm, vascular anomaly, endovascular embolisation

## Abstract

While isolated internal iliac artery aneurysms (IIIAAs) are rare entities, they are associated with a high mortality rate if ruptured. IIAAs are usually asymptomatic and can be discovered accidentally during imaging for any other causes. The treatment options vary according to the signs, symptoms, size of the aneurysm, and the patient’s general condition. While surgery used to be the first option of treatment earlier, with the advances in the field of endovascular intervention, endovascular repair of IIIAA has emerged as the first option of treatment if applicable.

## Introduction

An isolated internal iliac artery aneurysm (IIIAA) is defined as a two-fold increase in the size of the artery with at least a 50% increase in the diameter of the artery compared to the normal artery without a coexisting aneurysm in another location [1]. Iliac artery aneurysms usually manifest in conjunction with aneurysms of the abdominal aorta, with an incidence of approximately 10%, but isolated iliac artery aneurysms are rare and occur in only 2% of cases. An IIIAA is even more uncommon with an incidence of 0.4% [2]. They are more common in males and progress in size with aging. They may be asymptomatic and can be fatal if ruptured [3]. In this report, we discuss a case of isolated right internal iliac artery aneurysm managed effectively with endovascular embolization with an excellent outcome.

## Case presentation

**Figure 1:**
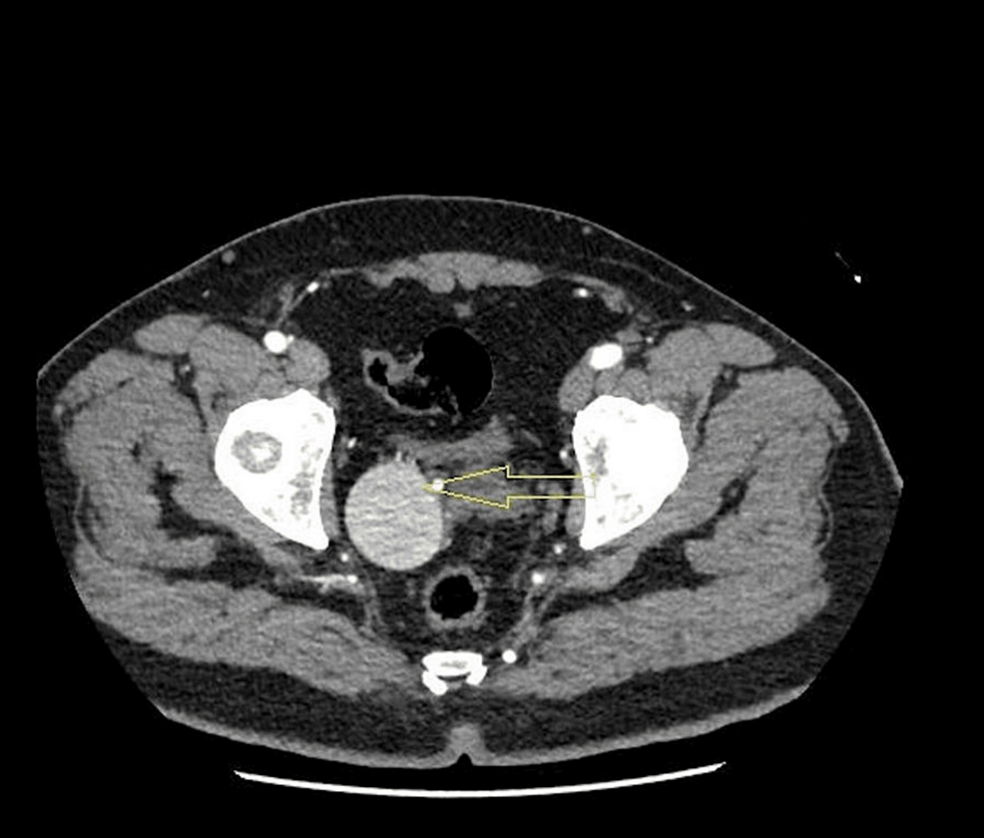
CT angiography The yellow arrow shows a pear-shaped aneurysm measuring 6.5 x 3.5 cm at the right-side pelvic cavity. The aneurysm can be seen displacing the adjacent urinary bladder seminal vesicles and prostate CT: computed tomography

**Figure 2:**
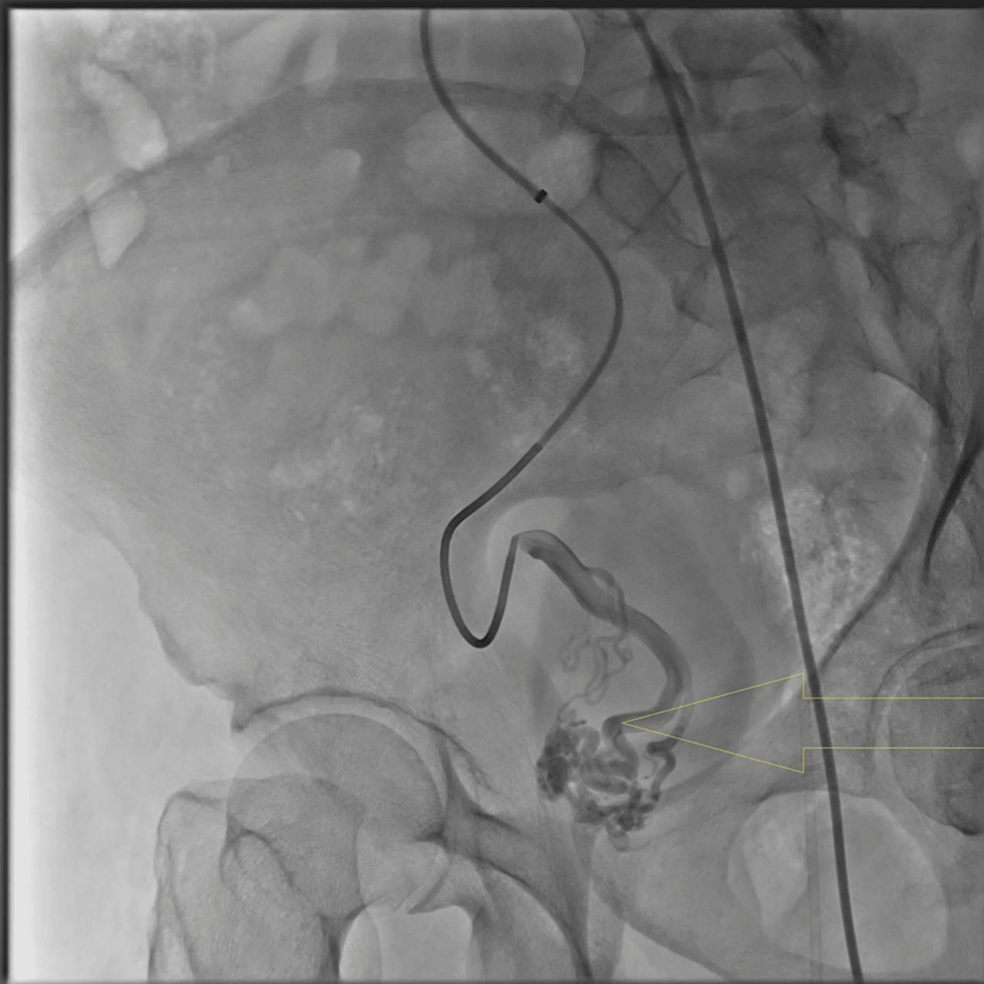
Diagnostic angiogram The yellow arrow shows multiple feeders coming over the right internal iliac artery aneurysmal dilation

A 46-year-old male from Jordan with no significant medical or surgical history presented to the clinic with right flank pain. A CT abdomen revealed an isolated internal iliac artery aneurysm. Further investigations including CT angiography and diagnostic cystoscopy were performed. CT angio showed a pear-shaped aneurysm measuring about 6.5 x 3.5 cm in the right-side pelvic cavity and supplied by branches from an internal iliac artery (Figure 1). The patient was admitted to our facility and consent was obtained for endovascular embolization of the IIIAA. Under local anesthesia, the left common femoral artery retrograde access diagnostic angiography showed multiple feeders coming over the right internal iliac artery aneurysmal dilation (Figure 2).

**Figure 3:**
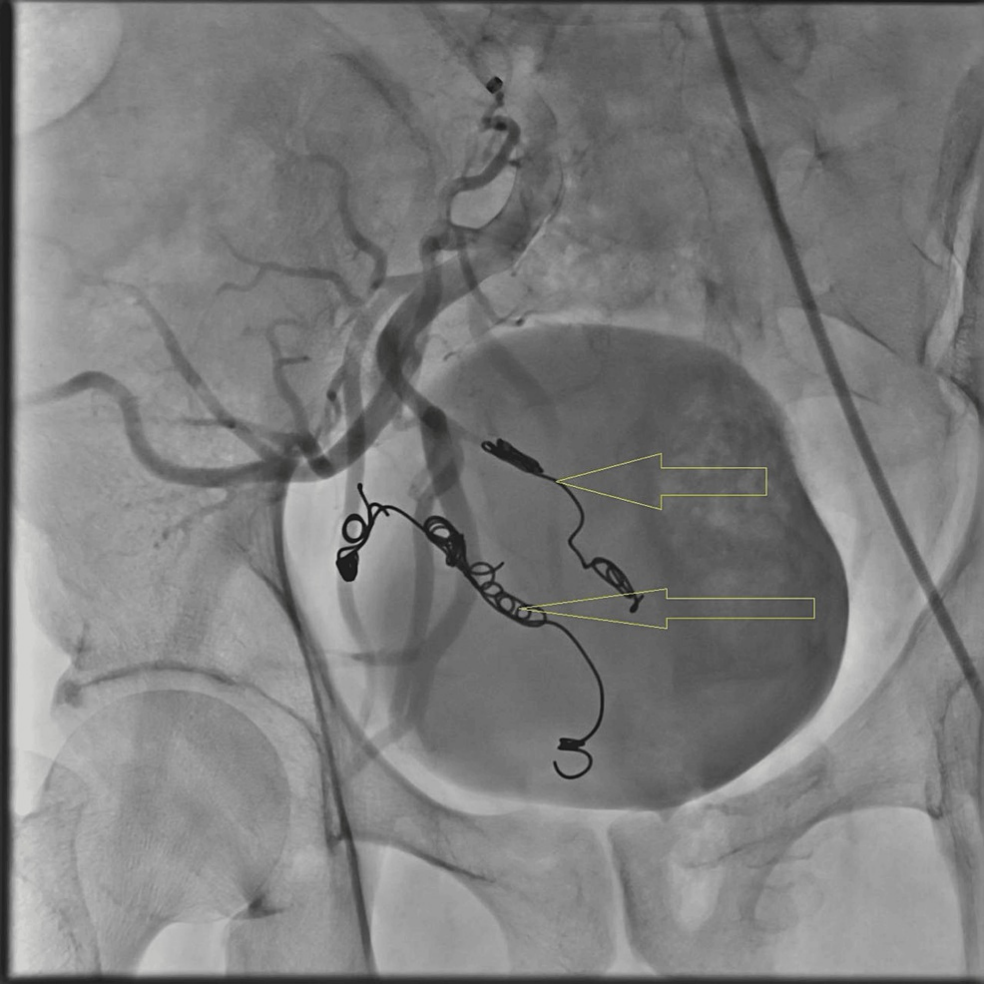
Angiogram The yellow arrows show post-coil embolization of the two hypogastric feeders of the right internal iliac artery aneurysm

**Figure 4:**
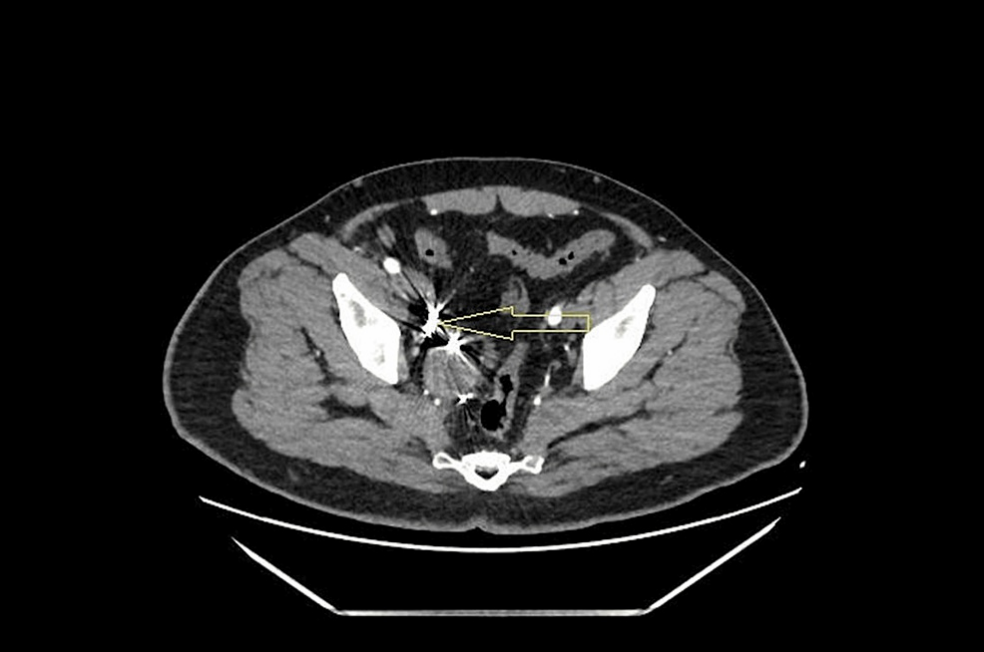
CT angio at the three-month follow-up The yellow arrow shows an aneurysm smaller in size (5.2 x 4.7 mm) with blood flow inside not enhancing with multiple small artifacts at its small narrow neck (indicating successful embolization) CT: computed tomography

The patient underwent endovascular coil embolization of the hypogastric branches feeding the aneurysm, resulting in successful on-table thrombosis (Figure 3). He had an uneventful operative and postoperative course and was discharged the same day. The patient came to the clinic one week after the procedure; he was doing well with no flank pain, no access-site complications, and no complaints of erectile dysfunction. A CT angio follow-up three months after embolization showed a smaller-sized aneurysm (5.2 x 4.7 mm) with blood flow inside not enhancing with multiple small artifacts at its narrow neck, indicating successful embolization of the right IIAA (Figure 4).

## Discussion

IIIAA refers to the abnormal dilatation of the blood vessel wall that usually occurs in combination with aortic aneurysms. Isolated internal iliac artery aneurysms are rare and can lead to serious complications if not diagnosed and treated properly; it is defined as a two-fold increase in the size of the artery without a coexisting dilation in any other artery [3]. The causes of IIIAA include atherosclerosis, infection, trauma, and vessel wall disorders. While most patients with IIIAA are elderly males, it can occur in a wide age range; it is more common in males than females [4]. An IIIAA may be asymptomatic at diagnosis, present with frank rupture, or demonstrate symptoms caused by the pressure on the adjacent organs [2].

The diagnosis of the condition is often challenging, and a rupture may be its initial presentation, leading to severe consequences. CT plays an important role in the diagnosis of intrabdominal aneurysms as it detects site, size, and relation to another organ and is used routinely before elective repair. A diagnostic femoral angiogram can be used as well and could identify arteriovenous fistula in the operating room [5]. To choose an appropriate treatment modality - surgery, which is technically difficult, or endovascular repair - several factors need to be taken into account, such as aneurysm anatomy, diameter, tortuosity, angulation of vessels, other associated aneurysmal diseases, urgency of repair, and patient comorbidities. Treatment options for IIIAA include ligation, excision, endoaneurysmorrhaphy, coil embolization, and endoluminal stenting. Endovascular embolization and coiling have proven to be effective and safe methods for treating isolated IIIAA [6].

## Conclusions

IIIAAs pose a significant challenge in diagnosis and treatment. Endovascular embolization and coiling are effective and safe methods for managing IIIAA, especially in cases with a high risk of rupture. An alternative for fit patients with challenging anatomy is still the open resection. This case report highlights the successful outcome of the endovascular management of a patient with an IIIAA.
